# Biomarker identification of hepatocellular carcinoma using a methodical literature mining strategy

**DOI:** 10.1093/database/bax082

**Published:** 2017-12-08

**Authors:** Nai-Wen Chang, Hong-Jie Dai, Yung-Yu Shih, Chi-Yang Wu, Mira Anne C Dela Rosa, Rofeamor P Obena, Yu-Ju Chen, Wen-Lian Hsu, Yen-Jen Oyang

**Affiliations:** 1 Graduate Institute of Biomedical Electronics and Bioinformatics, National Taiwan University, Taipei, Taiwan; 2 Institute of Information Science, Academia Sinica, Taipei, Taiwan; 3 Department of Computer Science and Information Engineering, National Taitung University, Taitung, Taiwan; 4 Interdisciplinary Program of Green and Information Technology, National Taitung University, Taitung, Taiwan; 5 Institute of Chemistry, Academia Sinica, Taipei, Taiwan

## Abstract

Hepatocellular carcinoma (HCC), one of the most common causes of cancer-related deaths, carries a 5-year survival rate of 18%, underscoring the need for robust biomarkers. In spite of the increased availability of HCC related literatures, many of the promising biomarkers reported have not been validated for clinical use. To narrow down the wide range of possible biomarkers for further clinical validation, bioinformaticians need to sort them out using information provided in published works. Biomedical text mining is an automated way to obtain information of interest within the massive collection of biomedical knowledge, thus enabling extraction of data for biomarkers associated with certain diseases. This method can significantly reduce both the time and effort spent on studying important maladies such as liver diseases. Herein, we report a text mining-aided curation pipeline to identify potential biomarkers for liver cancer. The curation pipeline integrates PubMed E-Utilities to collect abstracts from PubMed and recognize several types of named entities by machine learning-based and pattern-based methods. Genes/proteins from evidential sentences were classified as candidate biomarkers using a convolutional neural network. Lastly, extracted biomarkers were ranked depending on several criteria, such as the frequency of keywords and articles and the journal impact factor, and then integrated into a meaningful list for bioinformaticians. Based on the developed pipeline, we constructed MarkerHub, which contains 2128 candidate biomarkers extracted from PubMed publications from 2008 to 2017.

**Database URL**: http://markerhub.iis.sinica.edu.tw

## Introduction

Hepatocellular carcinoma (HCC) is a worldwide health issue, ranking fifth among all cancers and third among cancer-related deaths (1). With an overall 5-year survival rate of 18% (2), effective treatment of HCC relies upon the diagnosis of HCC at early stage, stressing the importance of robust screening tests. Currently, the most commonly used surveillance tests for HCC without pathologic confirmation are serum alpha-fetoprotein (AFP) and imaging tools, such as hepatic ultrasound, magnetic resonance imaging and computerized tomography (3, 4). However, these methods diagnose only 44% of patients at a localized disease stage, and only 30% of patients diagnosed with HCC qualify for curative treatments at the time of diagnosis (5). Thus, it is imperative to improve these tools by exploring potential effective biomarkers to increase the number of patients qualified for curative treatment and improve the HCC patients’ prognosis.

A biomarker is defined as a ‘characteristic that is objectively measured and evaluated as an indicator of normal biological processes, pathogenic processes, or pharmacologic responses to therapeutic intervention’ (6), which may be DNA, RNA, microRNA, protein or metabolites (7–9). In clinical settings, biomarkers are useful in disease screening, diagnosis and therapy, as well as monitoring its recurrence (10). In recent years, major investments have been made to develop biomarkers for major diseases. A large number of molecular biomarkers have been discovered, which lead to a parallel surge in electronic data availability (11). As a result, numerous scientific literatures on different diseases and their molecular mechanisms have been published.

In spite of the increased availability of scientific literatures via cutting-edge technologies, many of the promising biomarkers reported have not been validated for clinical use (12). To date, only the HE4 protein has been approved by the Food and Drug Administration as a biomarker for ovarian cancer in 2009 (13). In order to efficiently select candidate biomarkers for future clinical validation, bioinformaticians need to identify molecular biomarkers from information that has already been published (14). However, as the number of biomedical literatures grows, the difficulty and time required to evaluate potential biomarkers from these sources also increase without utilizing text mining tools.

Text mining is the development and use of computerized means to retrieve knowledge accessible from a wide range of information repositories (15). Applying text mining to extract information from biomedical and molecular literatures has been used to identify and search for interactions between disease-associated biological units, conceive hypotheses from available data and chart biological conduits (16). In addition, several text mining approaches have been proposed for biomarker extraction (17–20). In this study, we integrated machine learning approaches, including conditional random fields (CRFs) and convolutional neural networks (CNNs), and pattern-based approaches into a pipeline to automatically extract potential biomarker information from a collection of scientific literatures and generate a ranked biomarker list composed of genes. As a proof-of-concept, we used the developed pipeline to mine biomarkers for HCC and constructed our HCC-biomarker database named MarkerHub. The pipeline automatically recognizes several biological terms in the collected documents, such as genes, mutation information, cell lines and diseases. When a sentence contains genes recognized by the pipeline, a sentence classifier based on CNN is used to determine whether the sentence provided sufficient evidence to support the recognized gene/protein as a biomarker. Lastly, candidate biomarkers are stored in MarkerHub and ranked by a global ranking algorithm, which considers different ranking factors that capture the importance, relevance and novelty of the curated biomarkers. Furthermore, MarkerHub provides a network visualization tool that facilitates bioinformaticians in discovering novel associations between genes and diseases based on direct/neighborhood associations in the visualized network.

## Related work

The interactions between organisms and the environment constitute a huge amount of the existing biomedical relations. Identification of underlying relations between several genes and disease phenotypes is useful for doctors and researchers, and has been the topic of interest in several studies. Among these relations, protein–protein interactions were used to predict gene–disease relationships (21–23). Some studies determined the relationship by computing the similarity values between genes and diseases based on Gene Ontology (24) or Disease Ontology terms (7–9). Other controlled vocabularies such as MeSH have already been utilized for linking proteins to disease terminologies (25). Additional information such as gene expressions (26), protein/genome sequences (27, 28) and positional information (29) also serve as important evidence to substantiate the relationship between genes and diseases.

Furthermore, text mining techniques have been employed in an attempt to automatically extract gene–disease associations from biomedical literatures to construct gene–disease association (GDA) databases (30). To the best of our knowledge, most well-known text-mined GDA databases were developed based on two major strategies. Traditional rule-based methods focused on limited linguistic contexts and relied on word co-occurrences and pattern matching. For example, Bauer-Mehren et al. (30) proposed a knowledge-driven approach to extract biomedical named entities in scientific literatures. Based on the assumption that a biomarker and a disease are associated if they were mentioned together in the same sentence, 11% of the disease-biomarker associations identified by their approach were found in their database. Abul Seoud and Mabrouk (31) developed TMT-HCC to identify molecular biomarkers of HCC based on a pattern-based approach. The patterns were curated by domain exports for co-occurrence statistics. Alternatively, machine learning-based approaches were widely employed to extract GDAs (14, 32, 33). Singhal et al. (32) established a machine learning-based method to automatically distinguish mutations mentioned in biomedical literatures related to a particular disease. Their approach obtained F-measures of 0.880 and 0.845 for prostate and breast cancer mutations, respectively. Younesi et al. (14) built a biomarker information retrieval system by exploiting ProMiner (34) and SCAIView (35), with the output of the system being a ranked biomarker list. They found that the average coverage rate of six biomarker classes is 69.83% in a relevant text corpus. After extracting GDAs, network-based approaches can be employed to analyze the relationships among them (36–38). These works highlight the importance of GDAs as it can lead to a better understanding of diseases, which can facilitate the development of novel and effective drug therapies with less time and effort.

## Materials and methods

**Figure 1.**
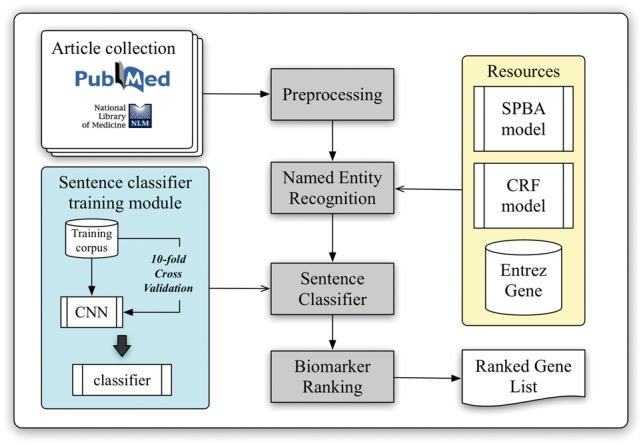
Schematic view of the developed curation pipeline.

The workflow of the developed biomarker extraction curation pipeline is shown in Figure 1. We elaborate each step in the following sub-sections.

### Article collection and classification

With the help of PubMed E-Utilities, articles related to liver cancer are collected from the PubMed database using the query ‘(((“Hepatocellular carcinoma”[Title/Abstract]) OR “Liver cancer”[Title/Abstract]) AND biomarker)’ without applying any language filters. Article metadata such as PMID, title, abstract, journal name and its ISSN and publication date are extracted. The journal information is used to verify the impact factor (IF) and the journal type from SJR (SCImago Journal & Country Rank), which will be used later in the pipeline for ranking. A total of three journal types were defined, including clinical research, translational research and basic research. In our implementation, the type of journal is determined by matching the journal name with a handcrafted journal name-type matching list manually compiled from SJR (The keyword list can be downloaded from our website.). Finally, all collected articles are preprocessed by several natural language processing components to extract linguistic information such as sentence boundaries, tokens and part-of-speech information.

### Named entity recognition

Although NCBI provides Pubtator (39), which delivers high-quality entity recognition of five common bio-concepts, biomedical entities like cell lines and miRNAs are not supported by their service. However, recognition of these entities is required for further biomarker scoring in our approach. Therefore, we adapted our BioC module (40) to identify gene and species mentions, and a pattern-based method based on the statistical principle-based approach (SPBA) (41) to recognize other biological concepts. The gene mention recognition task was formulated as a sequential labelling problem, and linear-chain CRFs were used to compute the probability associated with the corresponding hidden labelled sequence of a sentence. For the species terms, we scanned the entire article and partially matched the text with the species terms listed in a species dictionary, and then used full name-abbreviation information to extract the designated species symbol prefixed in a gene name. The details of the established algorithm are described in (40).

Moreover, we exploited the vocabularies defined by comparative toxicogenomics database (42), MeSH (43), Discovery Services (44), IGRhCellID (45) and HyperCLDB (46) to recognize chemical techniques, diseases and cell line entities. Additionally, we specified several keywords to distinguish mentions of mutation, statistical term, sample and concentration. For example, keywords used for statistical terms include ‘sensitivity’, ‘sensitivities’, ‘specificity’, ‘specificities’, ‘accuracies’, ‘accuracy’, ‘area under curve’, ‘AUC’ and the regular expression pattern ‘\d+%’. The pattern used for sample recognition was ‘\d + (sample(s)?|subject(s)?|case(s)?|patient(s)?|tumor(s)?){1}’, while ‘\d + (\.\d+)? (M|mM|uM|nM){1}’ and ‘\d + (\.\d+)? (g|mg|ug|ng){1}/(l|ml|ul){1}’ were used to label concentrations. All of these resources were exploited by SPBA to generate the principles used to match the content of the articles, thereby identifying the existing biomedical mentions. Detailed descriptions of SPBA can be found in our previous work (41).

### Sentence classification

**Figure 2.**
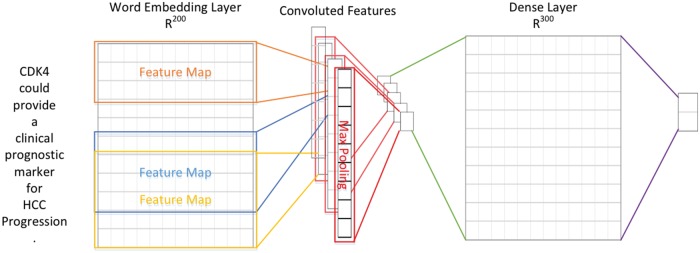
The CNN architecture developed for evidential sentence classification.

In order to extract supporting biomarker evidential sentences, we utilized the manually curated biomarker evidential sentences released by LiverCancerMarkerRIF (47) to construct a classifier. The task is formulated as a sentence classification problem in which a sentence is classified as evidential or not. A CNN was developed with one layer of convolution on top of a word embedding layer, which is followed by a fully connected layer and one softmax layer. Figure 2 shows the architecture of the model developed in this work, which is very similar to the CNN model used by (48, 49).

In contrast to computer vision where the input is usually a grid of pixels, the input layer of our CNN is a sequence of tokens. The tokens are generated by GENIATagger (50) for a given sentence. Each token in the input sequence is represented as a one-hot vector that indexes the token into a vocabulary. These vectors are mapped to low-dimensional representations through a word embedding layer initialized from the pre-trained word vectors released by Moen and Ananiadou (51) (The pre-trained word vector can be downloaded from http://evexdb.org/pmresources/vec-space-models/.). In our implementation, the size of the embedding vector was set to 200 dimensions, and the maximum length of a sequence was set to 100. The example sentence shown in Figure 2 is padded because its length is less than 100. Therefore, the 100 × 200 matrix can be considered as the ‘image’ for our CNN. The filters used in our model are w × 200 kernel matrixes, where w is the sliding window of 3–5 tokens, and there are ten filters for each sliding window. Finally, the convolutional layer is fully connected to a dense layer followed by a softmax layer for classification.

### Biomarker ranking

In our work, the ranking of curated biomarkers is determined by considering their importance, relevance and novelty. These characteristics are captured by: (i) The number of biomarker citations. (ii) The sum and average of the publications’ impact factors. (iii) The number of samples used in the study. (iv) The number of sentences containing both the target biomarker and statistical terms. (v) The biomarker score determined based on the results of article-level and sentence-level context analyses. The first factor quantifies the importance and the impact of a published paper. We assume that biomarkers being mentioned and discussed in more publications are relatively more important and well-investigated. It was observed that the majority of the biomarkers published in journals with high impact factors were more frequently cited, implying that they are of greater significance. Therefore, we defined the impact factor as the second factor to indicate the relevance and impact of the biomarker.

The third factor emphasizes the importance of a large sample size in biomedical studies. We assume that a study with a large sample size would better represent the population. The fourth factor measures the accuracy of the gene/protein that is studied by observing its related statistical terms. Finally, the last factor ranks the biomarkers at both the article-level and sentence-level depending on different parameters such as species and sample source. The ranking scheme of the last factor is elucidated in the following paragraphs.

The complete scoring strategies of article-level and sentence-level analyses are shown in Tables 1 and 2, respectively. The article-level scoring is divided into five categories: species, reference count, source, location and publication year. Under each category, there are two or more criteria with different weighted scores. Due to the complex nature of this task, we could not devise an effective automatic strategy for weight learning. Instead, weights for each criterion were assigned based on our expert‘s perspective. We assigned higher weights for genes mentioned in articles that involved humans or clinical specimens. For example, for a candidate gene biomarker, we assigned 5 points for each article if the biomarker was found in a study involving human species, one point for species other than human, or six points if both were mentioned. The rationale is that biomarkers discovered from humans and clinical specimens would more likely represent the human population rather than those involving other species or cell lines. In addition, a higher score is given for genes/proteins that are mentioned in the title as these may be the major biomarkers being studied or compared to other genes/proteins that exist in the body of the manuscript.

**Table 1.** Biomarker scoring at the article-level

Category	Score	Description
Species	5	Human
	1	Other Species
	6	Human + Other Species
Numbers of References	2	Count > 150
	1.5	50 < Count ≦150
	1	10 < Count ≦50
	0.5	Count ≦10
Source	5	The source of the biomarker is from Patient Sample,
	1	Cell Line, or
	6	Patient Sample + Cell Line
Location	5	The biomarker is described in Title or
	1	Abstract
Publication Year	3	≧2008
	1	≦2008

**Table 2.** Biomarker scoring at the sentence-level

Category	Score	Description
Disease Sentence	1	If the sentence contains the target biomarker and a disease mention.
Concentration Sentence	5	If the sentence contains the target biomarker and a concentration concept.

The second level of biomarker scoring is the sentence-level analysis, in which we observe whether the sentence contains the disease name and quantitative information on the genes/proteins and set the corresponding weighted scores (Table 2). Notably, quantitative information (concentration) may indicate differential expression of the gene/protein between different disease states. After calculating the article-level and sentence-level scores for a biomarker, both are aggregated to obtain the total biomarker score.

In order to rank the extracted biomarkers based on all of the ranking factors, a fusion-based global ranking framework proposed in our previous work (52) is implemented. Under this framework, the ranking factors were transformed into five ranking functions used by five ranking models. Let $x=\left\{x_{1},\;x_{2},\ldots ,\;x_{n}|\}\right\}$ denote the biomarkers curated by our pipeline. Each model produces its ranking score for *x*. Here we use $y_{i}=\left\{y_{1,\;i},\;y_{2,\;i},\ldots ,\;y_{n,i}|\}\right\}$ to denote the ranking scores assigned by the *i*th ranking model to *n* biomarkers. Note that for a ranking model *i* and a biomarker *x_k_*, *y_k, i_* may be zero if the biomarker does not possess the characteristic represented by the ranking model *i*. For instance, consider the fourth ranking factor in which we requested that the target biomarker *x_k_* must co-occur with a statistical term within one sentence. If *x_k_* does not meet this requirement, its score *y_k_*_, 4_ will be zero.

Based on the five ranking scores *y*_1_ to *y*_5_ generated by our five ranking models, we adapt the linear combination model (LC) fusion algorithm to aggregate their scores. Assuming that we have l individual ranking models, the LC ranking model calculates the ranking score ρ of $x_{k}$ against all ranking models as follows:

$$\rho \left(w,x_{k}\right)=\sum_{i=1}^{l\;}w_{i}y_{k,i}$$

where $w=\left(w_{1},\;w_{2},\;w_{3},\ldots ,\;w_{l}\right)$ represent the weights for the *l* individual ranking models. This score ρ is then used to rank all of the curated biomarkers. In our framework, the ranking score ρ can be calculated as follows:

$$\rho \left(w_{1},w_{2},w_{3},w_{4},w_{5},x_{k}\right)=w_{1}y_{k,1}+w_{2}y_{k,2}+w_{3}y_{k,3}+w_{4}y_{k,4}+w_{5}y_{k,5}$$

### Network construction

QuasiPro (http://csb2.ym.edu.tw/quasipro/index.php) is an online tool that collects protein–protein/gene–gene interactions (PPIs/GGIs) from several public databases with data mining approaches. We submitted all of the extracted gene/protein biomarkers to QuasiPro to generate full and directly interacting networks and plotted the networks with Cytoscape (http://www.cytoscape.org/).

## Results and discussion

### Results of biomarker curation

In this work, a total of 2128 gene/protein biomarkers were extracted from 12 052 articles related to liver cancer from PubMed. After preprocessing, a total of 58 874 sentences were generated. With the built CNN model for sentence classification, we filtered out 19 367 non-supporting evidential sentences. Table 3 summarizes the top 10 of the extracted biomarkers ranked by the five factors.

**Table 3.** Top 10 gene/protein biomarkers obtained from the developed curation pipeline

Gene(ID)	Article	Impact Factor	Statistics	Sample	Weight	Final Ranks
AFP(174)	649	4.48	220	62	207.57	1
F2(2147)	300	3.97	180	52	106.01	2
CEACAM5(1048)	200	3.75	77	36	61.81	3
TP53(7157)	169	4.16	41	6	56.98	4
CD8A(925)	171	5.36	46	1	57.58	5
EPCAM(4072)	96	4.24	36	2	31.44	6
GOLM1(51280)	73	3.43	40	37	18.14	6
FAM126A(84668)	84	4.03	41	7	28.49	6
IFNA1(3439)	86	4.57	31	1	27.86	7
IFNA13(3447)	86	4.57	31	1	27.86	7
TGFB1(7040)	93	4.31	21	2	31.46	7
PROM1(8842)	100	4.88	20	0	34.39	8
CDH1(999)	59	4.67	16	0	19.54	9
CHRNA1(1134)	43	2.72	29	12	14.32	9
VEGFA(7422)	57	3.47	17	1	18.69	9
AKT1(207)	102	5.37	10	1	36.20	10
CD4(920)	55	3.81	18	0	17.90	10
IL6(3569)	61	5.26	11	1	20.71	10
IL7(3574)	53	4.5	18	0	17.06	10
MKI67(4288)	55	3.09	25	0	19.23	10

As shown in Table 3, the top 5 candidate biomarkers are AFP, F2, CEACAM5, TP53 and CD8A. We conducted literature review to discuss their importance and potential as clinical biomarkers in the following subsections.

#### Alpha-fetoprotein

AFP is the serum biomarker widely used to test for HCC, but its sensitivity of 41–65% and specificity of 80–90% when detecting HCC at the cut-off value of 20 ng/mL is unsatisfactory (1). Because of its non-specificity, AFP is not suitable as the sole indicator to screen and diagnose HCC. Meanwhile, the LCA-reactive fraction of AFP (AFP-L3) has been reported to be a more accurate marker for HCC compared to AFP. Reports on AFP-L3 as an early diagnosis marker were published as early as 1993, where it was found that 73% of the patients with elevated AFP-L3 eventually developed HCC after 35 months (53). AFP-L3 has already been used in Japan for screening and diagnosing HCC (54).

#### F2 (Protein name: PIVKA-II or DCP)

DCP, also known as PIVKA-II, is an abnormal prothrombin discovered in 1984. In a previous study, plasma DCP was detected in 54.3% of the 628 patients studied, including 253 liver cirrhosis patients and 116 HCC patients. A positive correlation between its plasma concentration and tumor size was also observed (55). Another study revealed that DCP had a sensitivity of 52.8% and specificity of 98.8%, which are comparable to those of AFP. In 50 patients with HCC, the combination of AFP and DCP was found to be superior to using either AFP or DCP alone for diagnosis (56).

#### CEACAM5

CEACAM5 is the gene that encodes Carcinoembyronic antigen (CEA), a cell surface glycoprotein used as a marker for gastrointestinal cancers. As a cell adhesion molecule, it is considered to play an important role in tumor development, as well as the regulation of differentiation, apoptosis and cell polarity (57). In a recent report, CEA, along with 6 other proteins, were combined into a multimarker panel for primary HCC to acquire an overall improvement in sensitivity, specificity, accuracy rate and area under ROC curve of 82.0, 95.0, 90.1% and 0.884, respectively (58). In a 12-year study in Taiwan, an 8-marker panel including CEA identified common malignancies (including liver cancer, lung cancer, prostate cancer and colorectal cancer) with an especially high sensitivity for liver cancer at 90% (59).

#### TP53

Tumor protein 53 participates in a number of regulatory processes and induces apoptopsis, cell cycle arrest and metabolic changes. Mutations in the gene encoding this protein are closely correlated with various cancers (60). In a 2016 meta-analysis study, TP53 was found to be hypermethylated in patients with HCC. Hence, it was speculated that aberrant DNA methylations may be useful predictive and diagnostic markers for HCC (61). TP53 mutations were detected in 30–50% of HCC cases and found to be correlated to poor prognosis (62). Above all, it has been demonstrated that p53 mutations and overexpressions may serve as molecular prognostic factors for HCC (63).

#### CD8

CD8 is a glycoprotein found on the surface of cytotoxic T lymphocytes. In a recent study, low CD8+TIL count was found to be a predictor of poor HCC-specific survival in two independent cohorts. In combination with PD-L1 and Gal-9 expression, multivariate analysis revealed that this multimarker panel can be used to predict the survival of HCC patients (*P* < 0.001, HR 0.29, 95% CI 0.18–0.48) (64). The association of low CD8+TIL with poor survival is an indication of immune ignorance by tumors, as postulated by Teng and Smyth (65). Furthermore, in a prospective study involving 66 HCC patients that underwent surgical resection, low CD8 expression in distant non-neoplastic liver was correlated with high HCC recurrence rate (66).

### Validation of the curated list with other cancer biomarker databases

Aside from our work, there are a few publicly available cancer biomarker databases. Table 4 compares the coverage of MarkerHub with these databases. The first two are HCC-related databases: Liverome and MarkerRIF. The Liverome database (67) provides a comprehensive collection of well-curated HCC gene signatures from 98 HCC-related studies, including microarray and proteomic data. MarkerRIF (68) compiles a list of HCC-related genes and proteins from articles manually curated by users directly in PubMed. In addition, we compared MarkerHub with three other databases. (i) GeneCards Human Gene Database (69): a gene-centric database that combines information from large public sources including UniProtKB and provides concise genomic, proteomic, transcriptomic, disease and functional data. (ii) MalaCards (70, 71): an integrated database of human maladies and their annotations collected from 64 data sources. (iii) Catalogue of somatic mutations in cancer (COSMIC) Forbes et al. (72): a database that focuses on somatic mutations and chromosome abnormalities.

**Table 4.** Coverage of ranked candidate biomarkers with five on-line resources

	MarkerHub	Liverome	MarkerRIF	GeneCards	Malacards	COSMIC
# of genes	2128	6927	212	3485	165	3187
# of genes covered by MarkerHub	2128	1376	107	1206	108	1937
Coverage rate	100%	19.86%	50.47%	34.60%	65.45%	60.78%

On average, the percentage of the total gene records in the other five databases that were covered by MarkerHub is 33.9%. 50.47% of our previous work MarkerRIF was covered by MarkerHub, while Malacards and COSMIC had a coverage rate of 65.45 and 60.78%, respectively. Both GeneCards and Liverome obtained a coverage rate that is below 50%. MarkerRIF contained a total of 212 manually curated biomarkers that included non-human species such as *Drosophila*. Besides genes and proteins, miRNAs are also considered as a type of biomarker in MarkerRIF, while MarkerHub only focused on human genes in the liver. Among the 2128 biomarkers curated in MarkerHub, 107 human genes were found in MarkerRIF. Of the remaining 105 unique gene records in MarkerRIF, 54 human genes possessed lower gene expression profiles and 51 were non-human genes. By contrast, the rest of the unique gene records in MarkerHub were all found in liver with higher gene expression profiles in human sample according to the NCBI Gene Expression Omnibus database (73).

Genes in Liverome are manually curated from literatures with high-throughput assays. These assays can screen large-scale samples containing not only human genes, but also the genes of other species. However, it is difficult to validate the fidelity of nearly 7000 liver cancer-related genes. On the other hand, GeneCards curated both proteins and RNAs, and the latter in not included in MarkerHub. Therefore, the coverage rate is lower in Liverome and GeneCards in comparison to the others.

The effectiveness of the ranking algorithm is illustrated in Table 5. By manually cross-checking the top 30 genes associated with ‘Hepatocellular Carcinoma’ in each of the databases, we can observe that applying only a single ranking scheme resulted in a lower coverage (59–70%). For example, the coverage rates of applying single ranking schemes individually against the COSMIC database are only 67–80%. After employing the proposed global ranking scheme, the coverage rate can be improved to 90% for GeneCards and 87% for COSMIC, respectively. The results demonstrate the advantage of implementing the global ranking method.

**Table 5.** Coverage of the top 30 biomarkers ranked by the global ranking algorithm

	Article	IF^a^	Sample	Statistics	Weight	Global Ranking
MarkerRIF	30/30=1.0	30/30=1.0	29/29=1.0	30/30=1.0	29/29=1.0	30/30=1.0
Liverome	30/30=1.0	30/30=1.0	29/29=1.0	30/30=1.0	29/29=1.0	30/30=1.0
GeneCards	20/30=0.67	21/30=0.7	18/29=0.62	19/30=0.63	17/29=0.59	27/30=0.9
COSMIC	20/30=0.67	24/30=0.8	19/29=0.66	20/30=0.67	18/29=0.62	26/30=0.87
Average Coverage	0.835	0.875	0.82	0.825	0.8025	0.9425

a IF = journal impact factor.

### Network analysis

The results of validation indicate that many of the top-ranked biomarkers were also listed in the other online databases. However, these databases do not elucidate the association index or relative ranking of biomarker genes with respect to HCC. Information as such can only be examined through wet-lab data or cross-referencing the citation number and other attributes from these databases against our filtering parameters. To substantiate the credibility of HCC biomarkers in MarkerHub, we conducted network analysis to investigate the functional relations between proteins and validate disease-associated genes.

**Figure 3.**
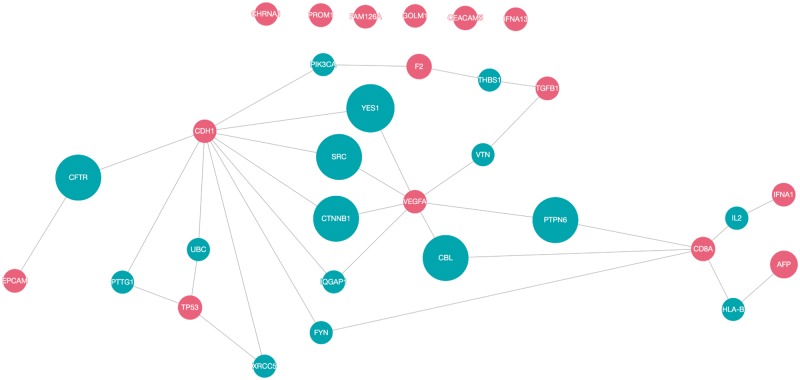
Network pathway analysis of top 15 proteins (red).

To determine the possible functional relations of the biomarker candidates, protein interaction networks of the top 15 proteins were constructed using QuasiPro (http://csb2.ym.edu.tw/quasipro/index.php). QuasiPro is an online tool which collects protein–protein/gene–gene interactions (PPIs/GGIs) from several public databases through data mining approaches. Using this tool, we constructed both full and direct connection networks, which consisted of the 31 curated genes/proteins (Figure 3).

It has been previously suggested that proteins with the same disease phenotypes are likely to be involved in the same signaling pathway or signal transduction mechanism (74). Thus, potential disease-associated genes or proteins can be derived from these PPIs (75). As shown in the network, there are direct interactions between 25 of the 31 proteins, signifying that these proteins are functionally related. The direct interactions uncovered between the majorities of the proteins extracted by our method supports the fact that they are indeed correlated to HCC.

Of the 31 proteins, CDH1 has the most interacting proteins with a total of 10 direct interactions, followed by VEGFA (7), CD8A (5), TP53 (4), F2 and TGFB1 (2 each), and EPCAM, AFP and IFNA1 (1 each). VEGFA and CD8A are also worthy of attention, since they are connected to two other protein interaction networks. CDH1, VEGFA, TP53, TGFB1 and EPCAM participated in pathways related to cell growth and proliferation and apoptosis (76). Dysregulated cell proliferation pathways and suppressed apoptoses commonly lead to uncontrollable proliferation of tumor (77). Moreover, according to the KEGG database (78–80), CDH1, TGFB1, TP53 and VEGFA were involved in pathways linked with cancer. Additionally, IFNA1, IFNA13 and TP53 were associated with pathways related to Hepatitis B and C, which are both high risk factors for hepatocellular carcinoma.

CDH1 is notable for regulating cell-cell adhesions, mobility and proliferation of epithelial cells (81). VEGF is an angiogenic factor that signals the central rate-limiting step in angiogenesis, which is critical in tumor formation and progression (82). As discussed previously, a number of evidences indicate that CD8 is involved in the process of T-cell mediated cytotoxicity. TP53 is a gene that commonly undergoes somatic mutations in human cancers, resulting in single amino-acid alterations at various positions (83).

It is worth noting that AFP, the FDA-approved serum marker for HCC (84), has only one interaction despite its high rank. It is connected to CD8A through HLA-B, which is a part of the immune regulatory functions (76). Produced in the yolk sac and in the liver during fetal development (85), AFP binds and transports bilirubin, fatty acids, retinoids, steroids, heavy metals, dyes, phytoestrogens, dioxin and various drugs (86). While AFP is known to be involved in liver development and organ regeneration (76), the role AFP plays in liver diseases is not yet fully understood, which may account for its meager participation in the constructed network.

### Web interface

**Figure 4.**
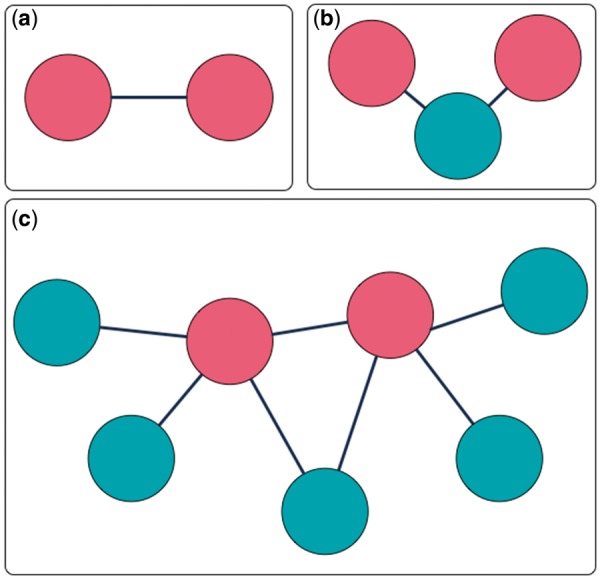
Three interaction extraction types: (**a**) direct interaction; (**b**) including connectors; (**c**) all neighbors.

MarkerHub provides various query options and a graphical visualization page to facilitate the access of network data of the ranked HCC biomarkers. Two major pages named ‘Markers’ and ‘Networks’ are included. The ‘Markers’ page shows the details of each ranked gene including the number of articles, the median of impact factors, statistical information, the number of samples, the weights and the global ranking result. On the other hand, the ‘Networks’ page presents the interactions of interest with three different modes of displays: (i) direct interaction; (ii) including connectors and (iii) all neighbors. First, the ‘direct interaction’ mode only shows the direct interactions among the selected biomarkers (Figure 4a). The ‘including connectors’ mode includes biomarkers that serve as connectors in between the selected biomarkers (Figure 4b). The ‘all neighbors’ mode exhibits all neighboring genes/proteins of the selected biomarkers (Figure 4c). Biomarkers can be selected by ticking the checkbox, and the corresponding networks would be generated based on the mode of network display.

### Performance of evidential sentence classification

We used the dataset released by LiverCancerMarkerRIF to develop our sentence classifier. The dataset contains 909 sentences manually annotated by the annotators recruited in the BioCreative IV user interactive task (87). Each sentence is annotated with a label indicating whether the sentence contains supporting evidence of liver cancer biomarkers. After tokenization, each sentence on average contains 26.7 tokens.

Table 6 compares the performance of the built CNN-based sentence classifier with four well-known machine-learning approaches including decision tree, support vector machine, Naïve Bayes and Naïve Bayes Multinomial based on a ten-fold cross validation on the dataset. The four algorithms were implemented by using Weka with default parameters and bag-of-word features (unigram-trigram). The results demonstrate that CNN outperformed the others by achieving a satisfactory F-score of 0.89.

**Table 6.** Performance comparison of different sentence classification models

Algorithm	F-score
Decision Tree	0.78
Support vector machine	0.85
NaïveBayes	0.82
NaïveBayesMultinomial	0.85
CNN	0.89

### Limitation

One of the concerns regarding the ranking scheme is the integration of the impact factor. Impact factor indicates the trend of articles being cited for a certain journal. Although this is not an absolute indicator of quality research, it provides a relative measure of the universality of the journal. Generally, journals with a higher impact factor receive more submissions and may set higher standards when reviewing them considering the reputation and broader audience of the journal itself. As there are no absolute factors to assess the quality of research, we adapted this measure as the score assigned to an article for ranking in combination with the other factors. However, as pointed out during the review of this work and the note given by Seglen (88), ‘Article citation rates determine the journal impact factor, not vice versa’, it may be problematic to consider the impact factor of the journal rather than the citation rate of an article when assessing its importance. In the future, we will incorporate the citation rate of each publication as a ranking factor in the ranking scheme.

## Conclusion

With the huge amount of available data from HCC clinical studies, a proper data curation and pipelined platform is required to help researchers retrieve potential biomarkers from existing literatures. In this work, we introduced a curation pipeline developed for mining biomarkers of HCC and constructed the MarkerHub database. The developed curation pipeline employs several state-of-the-art text mining components to extract biomarkers from a large collection of online literatures and implements a global ranking strategy with several ranking factors to sort the candidate biomarkers. The ultimate goal of biomarker-related studies is to come up with a panel of biomarkers for disease screening or monitoring. MarkerHub facilitates biomarker researches by providing life scientists with a ranked list that can be validated in a larger population using clinical specimens. Our ranked list is ideal for those employing or establishing multiplexed analysis tools like mass spectrometry or microarray. Depending on the multiplexing capability of the method, users can select the preferred number of genes/proteins from the ranked list for further investigations. In addition, a network analysis was included in MarkerHub to visualize the associations among the extracted biomarkers to assist researchers in acquiring a more comprehensive view of the potential roles these biomarkers may play in the progression of the disease. An interesting goal in the future would be to extract the evidence identified through the pipeline and prepare these data in the proper formats for statistical meta-analysis. On the whole, we believe that MarkerHub along with our curation pipeline can help life scientists by reducing the time and effort spent on article collection and data analysis. It is a useful and generic tool for mining biomarkers for various diseases, provided that the library and some key annotations are modified accordingly.
